# Comparison between two cell collecting methods for liquid-based brush biopsies: a consecutive and retrospective study

**DOI:** 10.1186/s12903-021-01557-5

**Published:** 2021-04-16

**Authors:** Kristin Gaida, Lena Deuerling, Heinrich Neumann, Torsten W. Remmerbach

**Affiliations:** 1 Department of Oral, Maxillofacial and Facial Plastic Surgery, Section of Clinical and Experimental Oral Medicine, Leipzig University Hospital, Liebigstraße 10-14, 04103 Leipzig, Germany; 2 Medical Care Centre for Histology, Cytology and Molecular Diagnostics, 52351 Düren, Germany

**Keywords:** Oral cancer, Squamous cell carcinoma, Brush biopsy, Liquid-based cytology

## Abstract

**Background:**

This study compares two different cell collectors, the Orcellex Brush (rigid brush) and the Cytobrush GT (nylon brush), using liquid-based cytology. A comparison of their obtainment procedures was also considered. The aim was to determine the diagnostic accuracy for detection of malignancy in oral brush biopsies. PICO-Statement: In this consecutive and retrospective study we had as population of interests, patients with oral lesions, the intervention was the brush biopsy with two different cell collectors and the control was healthy oral mucosa. The outcome of the study was to compare both cell collectors.

**Methods:**

From 2009 to 2018, 2018 patients with oral lesions were studied using the nylon brush (666 cases) and rigid brush (1352 cases). In the first cohort five smears per patient were taken with the nylon brush, while each patient received one smear with the rigid brush in the second cohort. These were further processed in a liquid-based procedure. Cytological evaluations were categorised into ‘negative’, which were considered as negative, whereas ‘doubtful’, ‘suspicious’ and ‘positive’ cytological results were overall considered as positive for malignancy in comparison to the final histological diagnoses. Additionally, the clinical expenditure for each collector was estimated.

**Results:**

2018 clinically and histologically proven diagnoses were established, including 181 cases of squamous cell carcinomas, 524 lichen, 454 leukoplakias, 34 erythroplakias and 825 other benign lesions. The sensitivity and specificity of the nylon brush was 93.8% (95% CI 91.6–95.5%) and 94.2% (95% CI 91.8–95.5%) respectively, whereas it was 95.6% (95% CI 94.4–96.6%) and 84.9% (95% CI 83.8–87.5%) for the rigid brush. The temporal advantage using the plastic brushes was 4×  higher in comparison to the nylon brush. The risk suffering from a malignant oral lesion when the result of the brushes was positive, suspicious, or doubtful was significantly high for both tests (nylon brush OR: 246.3; rigid brush OR: 121.5).

**Conclusions:**

Both systems have a similar sensitivity, although only the rigid brush achieved a satisfactory specificity. Additional methods, such as DNA image cytometry, should also be considered to improve the specificity. Furthermore, the rigid brush proved to be more effective at taking a sufficient number of cells, whilst also being quicker and presenting less stress for the patient.

## Background

Squamous cell carcinomas (SCCs) emanate from the surface of the multi-layered squamous epithelium. Among the malignant transformations in the mouth, jaw and facial areas, SCCs are the most frequent tumour type at 80% [1, 2]. Developing from a carcinoma in situ, SCCs histopathologically show an infiltrative growth beyond the epithelial basement membrane in the initial stage. In addition, small accumulations of atypical keratinocytes with a central ulcer, caused by ischemic areas and traumata, appear. This type of carcinoma grows endophytically or exophytically [3]. In 2020, the National Cancer Institute expected 53.260 new cases of cancer of the oral cavity and oropharynx and 10.750 deaths in the USA alone [4].

Oral SCCs should not be the only focus of clinicians, however, but also potentially malignant disorders (PMDs). According to the WHO (World Health Organisation 2017), a PMD is a clinically altered tissue with a high risk of developing cancer and includes a risk of cancer development in clinically normal mucosa [5]. PMDs include a number of diseases, e.g. leukoplakia, erythroplakia, lichen, forms of keratosis and palatal changes caused by smokeless tobacco or habits such as 'reverse smoking'. Leukoplakia, which can only be clinically diagnosed is a whitish, non-removable mucosal change that cannot be attributed to any other clinical disease [5]. The risk of malignancy increases depending on the morphology if the surface is reddish, erosive or even verrucous, inhomogeneously, it raises the risk of developing SCC. Erythroplakia is defined by the WHO as a reddish mucosal change that cannot be attributed to any other clinical disease and has a 17.5% higher risk of malignancy than homogeneous leukoplakia [5, 6].

The clinician screens the face, lips and mouth before examining changes in size, colour and texture. In addition, palpatory examinations are performed to diagnose changes in consistency and temperature of the mucosa, skin, bones, joints and lymph nodes [7]. If abnormalities appear, further examinations, such as the obtaining of cytological preparations for cytopathological examination, should be performed [8, 9]. In early diagnostics, the scalpel biopsy is still considered the gold standard but means discomfort for the patient [10]. It is recommended that the scalpel biopsy should be performed by a specialist, preferably the surgeon, who would later perform the surgical tumour therapy [11–15]. These limitations necessitated an alternative procedure that was accessible to most clinicians.

Between 2009 and 2012 we performed liquid-based cytology using the nylon brush. The cohort contained 666 specimens. This procedure was repeated five times and pooled in one vial upon the recommendation of the analysing cytopathologist. From 2012 to 2018 the in use diagnostic procedure was also liquid-based cytology but with the nylon brush. It was placed once and washed out in SurePath™ fixation liquid vials. This cohort contained 1352 specimens.

PICO-Statement: This paper presents a consecutive and retrospective study comparing the results from January 2009 to February 2018 in our special consultation hour for oral diseases. The outcome parameters were defined as followed: We determined the diagnostic accuracy for detection of histologically proven malignancy in comparison of two different types of cell collectors resp. obtainment procedures.

Additionally, we compared the total expenditure of both procedures per obtainment of a lesion.

## Materials and methods

This study represents a data collection on consecutive patient cases treated in the Dept. of Oral Maxillofacial and Facial Plastic Surgery, Section of Clinical and Experimental Oral Medicine, Leipzig University Hospital. The patients were examined anamnestically and clinically by qualified oral surgeons. Between 2009 and 2018, 2018 patient cases were enrolled and subsequently evaluated in this study. We have included patients with clearly visible oral lesions which showed apparent variation of the normal healthy mucosa. Overall, 2018 histologically proven final diagnoses could be stated: 181 squamous cell carcinomas, 524 diagnostically verified as lichen, 168 of them could be classified more precisely as erosive lichen. Oral leukoplakia occurred 454 times, 33 of which were considered to be proliferative verrucous type. 34 cell samples were counted as erythroplakia. The largest proportion of 825 oral lesions were classified as proven non-malignant lesions (Table 1).

**Table 1:** Summary of final diagnoses and their frequencies

Diagnoses	Frequency nylon brush		Frequency rigid brush	
	Absolute	Relative (%)	Absolute	Relative (%)
Squamous cell carcinoma	76	11.4	105	7.8
Oral lichen planus	96	14.4	260	19.2
Erosive lichen	30	4.5	138	10.2
Leukoplakia	124	18.6	297	22.0
Proliferative verrucous leukoplakia	13	2.0	20	1.5
Erythroplakia	5	0.8	29	2.1
Other non-malignant lesion	322	48.3	503	37.2
Total	666	100	1352	100

**Fig. 1:**
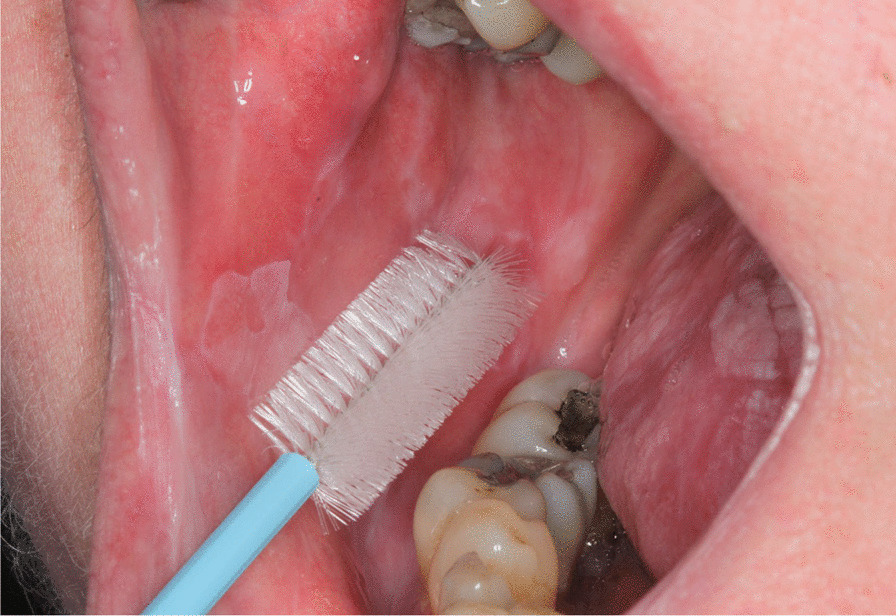
Exemplary presentation of a nylon-based cell collector in front of a plaquelike oral lichen

**Fig. 2:**
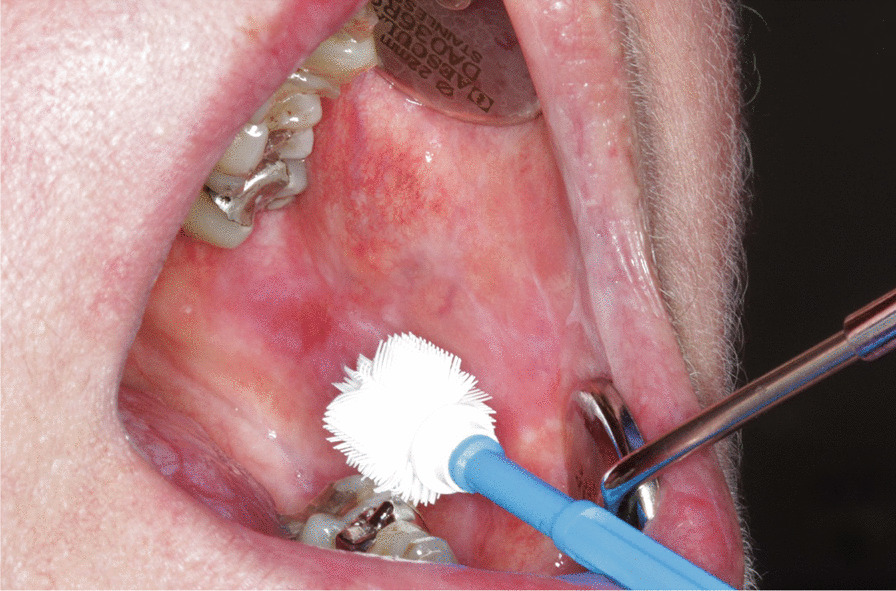
Plastic-based cell collector in front of an oral lichen

**Fig. 3:**
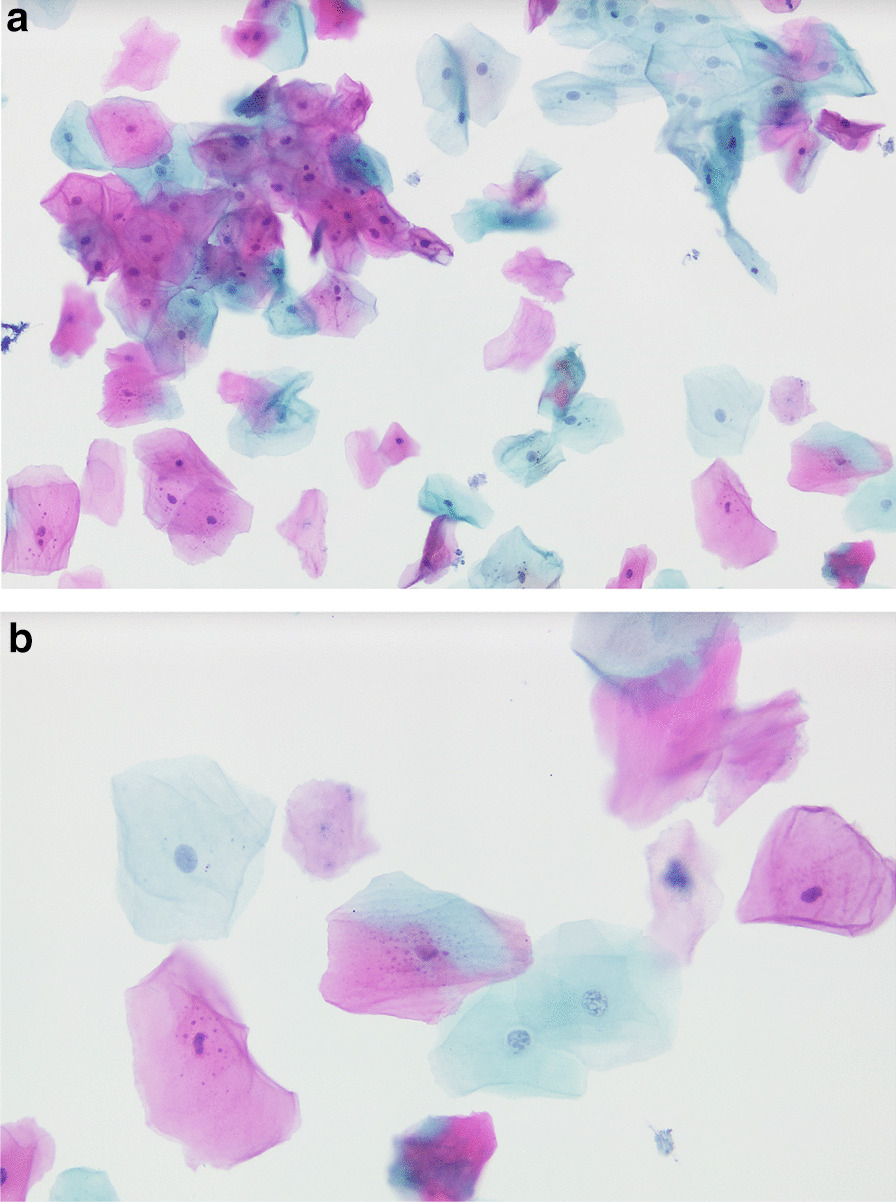
**a** SurePath™, staining Papanicolaou, lens 10x. Clinically: Follow-up of oral lichen planus. Cell-rich thin-layer preparation, mature squamous epithelia, individual nucleus less keratinized plaques, with tendency to hypereosinophilia, partly also amphophilia of the cytoplasm, individual hydrophilic swollen nuclei. Diagnosis: Malignant cells not present. **b** SurePath™, staining Papanicolaou, lens 40×. Clinically: Follow-up of oral lichen planus. Central a cell with partly eosinophilic, partly basophilic cytoplasm (amphophilia), nuclei of the two basophilic cells below are slightly enlarged with coarse chromatin as an expression of degenerative changes. Diagnosis: Malignant cells not present

**Fig. 4:**
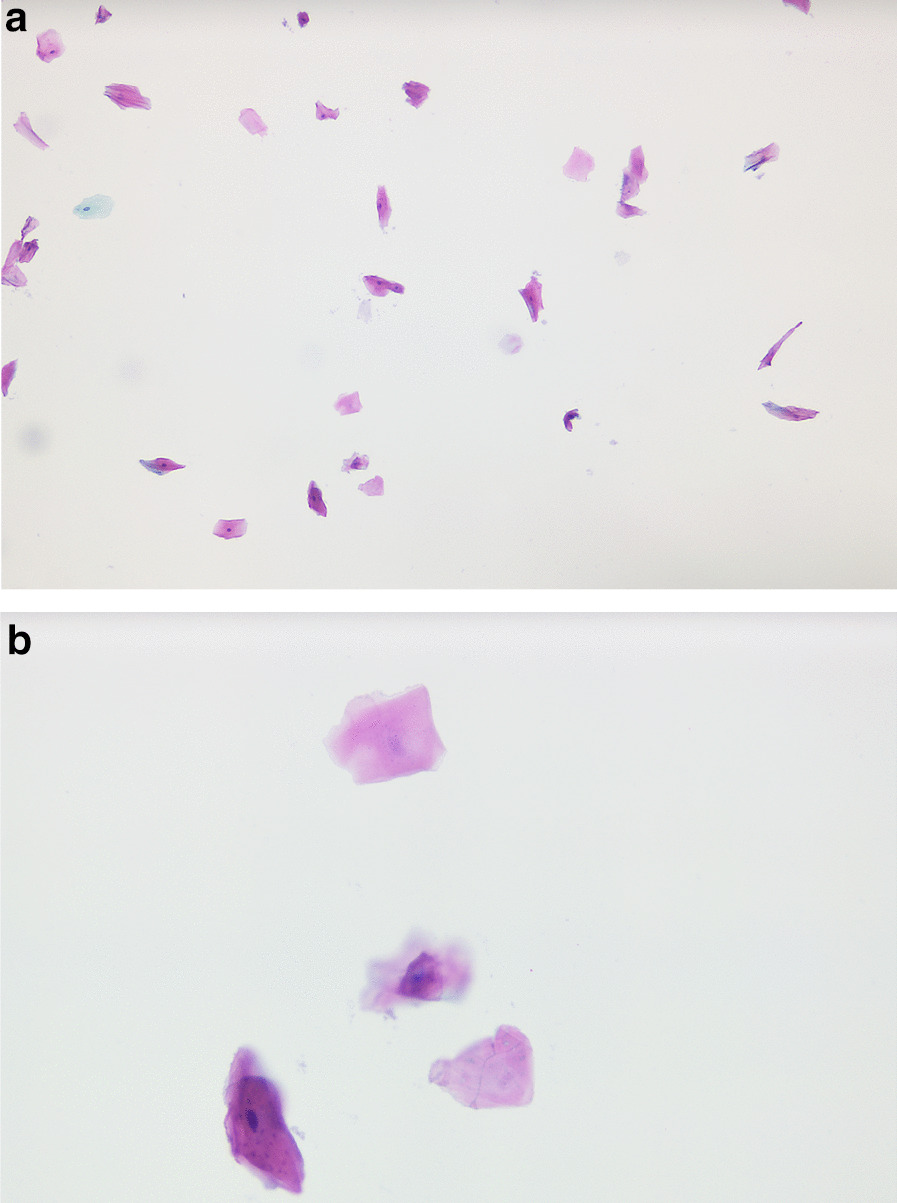
**a** SurePath™, staining Papanicolaou, lens 10x. Clinically: Whitish mucosal lesions on the tongue surface, suspicion of candida. Moderately cell-rich thin-layer preparation, mature squamous epithelia, numerous nucleusless keratinized plaques, clear background, no suspicious nuclear lesions, no signs of fungi. Diagnosis: Malignant cells not present. The increased tendency to keratinisation mainly indicates a leukoplakia. There are no signs of candidiasis. **b** SurePath™, staining Papanicolaou, lens 40x. Clinically: Whitish mucosal lesions on the tongue surface, suspicion of candida. At high zoom mainly cell-free keratinized plaques, visible nuclei are small, plain and unsuspicious. Diagnosis: Malignant cells not present. The increased tendency to keratinisation mainly indicates a leukoplakia. There are no signs of candidiasis

**Fig. 5:**
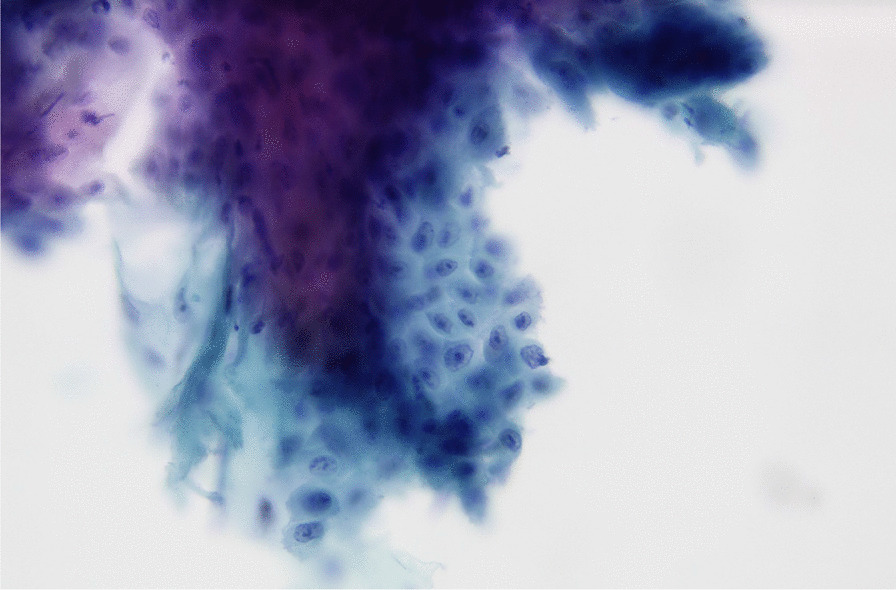
SurePath™, staining Papanicolaou, lens 40×. Clinical: an ulcerative lesion of the lateral border of the tongue. Immaturity, high nuclear/cytoplasmic ratio and anisonucleosis can be suspected in this magnification. The nuclei are haphazardly orientated, the axis of different nuclei is not parallel. Many nuclei show prominent nucleoli and/ or irregularities of the borders. Chromatin frequently is irregularly deposited with early condensation along the nuclear membrane. These changes may represent so called atypical tissue repair. The differential is high grade SIL or invasive SCC. The background may represent the ulcer or tumor diathesis. Diagnosis: Suspicious for malignant cells. We would try to confirm the suspicion with DNA-karyometry. A scalpel biopsy is strictly advised for further analysis

**Fig. 6:**
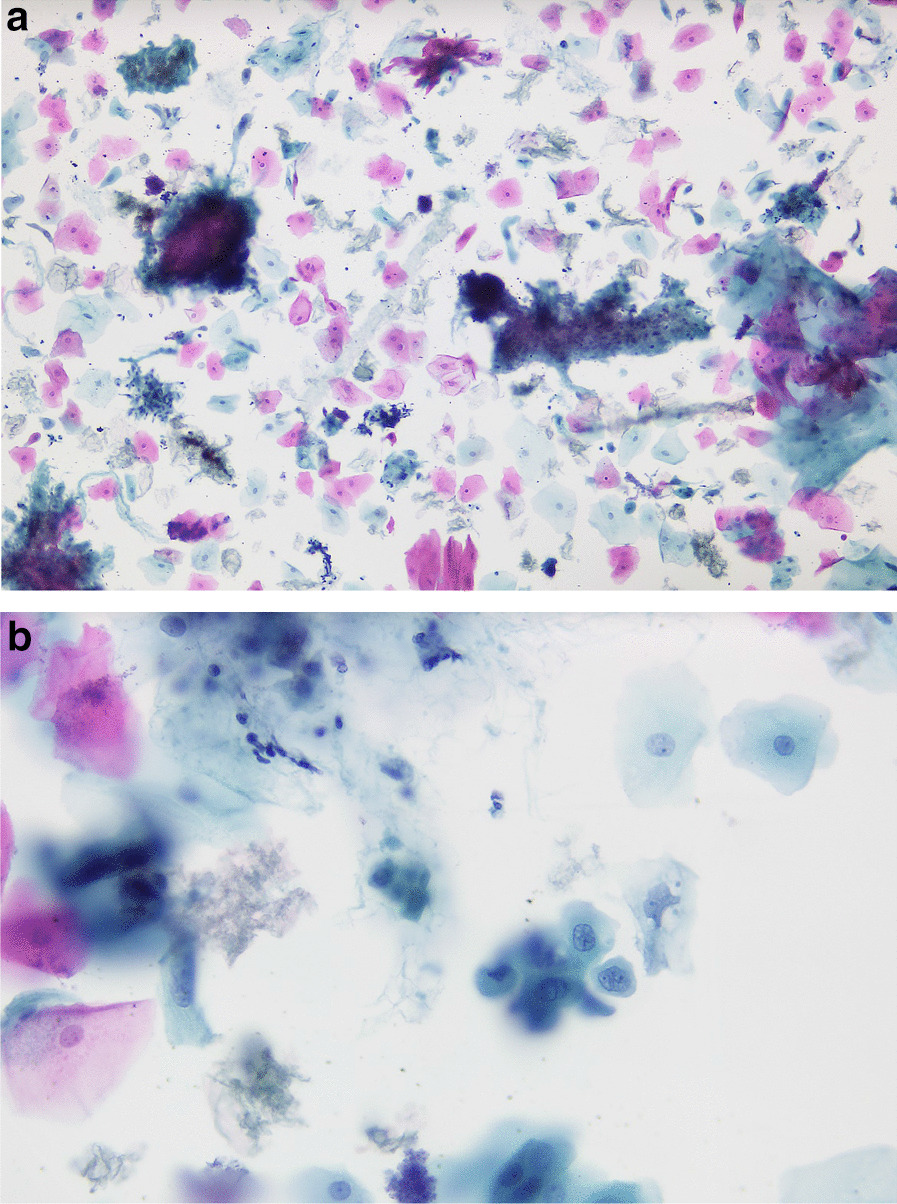
**a** SurePath™, staining Papanicolaou, lens 10x. Clinical: erosion of oral mucosa (mandible), condition after extraction 3 years ago, 1–2 packs of cigarettes daily. Cell-rich thin-layer preparation, mature squamous epithelia, some nucleusless keratinized plaques, plenty of fibrin and lysed blood in the background, individual squamous epithelia with shifted nucleus-plasma ratio and arranged in differently sized, partly two-, mostly three-dimensional clusters. Diagnosis: Malignant cells present. The cell picture corresponds to a keratinising squamous cell carcinoma. **b** SurePath™, staining Papanicolaou, lens 40x. Clinical: erosion of oral mucosa (mandible), condition after extraction 3 years ago, 1–2 packs of cigarettes daily. Fibrillary material in the background (tumor diathesis), small group of tumor cells with large, mostly deformed nuclei and some recognizable macronucleoli. Diagnosis: Malignant cells present. The cell picture corresponds to a keratinising squamous cell carcinoma

Between January 2009 and May 2012, the Cytobrush GT (Med-Scan Medical, Malmö, Sweden) was used for the sampling of conspicuous oral lesions (Fig. 1). The Orcellex Brush (Rovers Medical Devices B.V., Oss, The Netherlands) was used from June 2012 to February 2018 (Fig. 2). For both collectors, a liquid-based preparation method was used (SurePath™, BD Diagnostics, Tripath, USA). For this purpose, the clinician took at least five brush smears per lesion to be examined with the nylon brush and in comparison, only one obtainment using the rigid brush was performed routinely. The head of the brush was placed tangentially to the oral lesion and rotated at least ten times. Subsequently the head was washed out thoroughly in the preservation vial. The specimens were shipped to an experienced pathologist (HM). After arrival, all samples were further processed according to SurePath™ protocol (BD Diagnostics, Tripath, USA). The cells were deposited differently by gravitational force and then transferred to glass slides. Routinely the pathologist stained the cell samples according to Papanicolaou (PAP). The evaluation followed the German classification for extragenital cytology according to Böcking et al. [16]. This guideline showed four increasing categories of the probability of malignancy: If the result was 'negative', this meant that no malignant cells were detectable. If the examination revealed a 'doubtful' diagnosis, malignant cells may have been present and the existence of these could not be excluded. The pathologist noted 'suspicious' if cells with urgent suspicion were present, but malignancy was not definitely detectable. In contrast, malignant cells were clearly present when the pathological diagnosis was 'positive' (Figs. 3a, b, 4a, b, 5, and 6a, b). At the same time, there were also samples that could not be analysed. In this study, the results 'doubtful', 'suspicious' and 'positive' were summarized as 'tumour cell positive'. Only 'negative' was reported as 'tumour cell negative' for the statistical evaluation in comparison to the histological outcome as control group for both test groups [16].

## Patients

Overall, 2026 patient cases are documented. Eight of these cases could not be analysed cytologically due to insufficient sample quality. The average age of all patients was 61 years. The mean age of men was 58 years, of women 63 years. Among the 2026 patients 52% were female, 48% male (CI: 95%). The statistical evaluation was carried out by Stata (Version StataSE 15).

## Results

Looking at the results under this aspect, 405 statistically positive cytological diagnoses were available; the proportion of negatively assessed healthy patients was 1613.

Sensitivity was 93.8% (CI: 95%) for the nylon brush and the specificity achieved 94.2% (CI: 95%) (Table 2). The nylon brush detected a total of 54 lesions as cytological positive, but one sample turned out to be false positive. Of the 56 doubtful or suspicious cytological diagnoses, 33 were negative for malignancy in the final follow-up. Five of the 556 negative cytological diagnoses were proven to be false negative by histology (Table 3).

**Table 2:** Summary of diagnostic accuracy of brush biopsies detecting malignancy using different collectors and procedures

Diagnostic test	Accuracy		Upper endpoint (CI: 95%)		Lower endpoint (CI: 95%)	
	Rigid brush	Nylon brush	Rigid brush	Nylon brush	Rigid brush	Nylon brush
Sensitivity	95.60%	93.80%	0.967	0.955	0.945	0.916
Specificity	84.90%	94.20%	0.868	0.955	0.829	0.918
Positive predictive value	36.60%	69.10%	0.371	0.726	0.311	0.656
Negative predictive value	99.50%	99.10%	0.999	0.998	0.991	0.984
Odds ratio	121.5	246.3				
McNemar test (*χ*^2^)	170.6	20.10

**Table 3:** Modified 2 × 2 contingency table of brush biopsies using nylon brush compared to histology

Cytology Test: nylon brush	Histology					
	Positive for malignancy		Negative for malignancy		Total	
Positive	53		1		54	
Suspicious	17	76	3	34	20	110
Doubtful	6		30		36	
Negative	5		551		556	
Total	81		585		666	
Overall accuracy	94.10%					

The sensitivity of the rigid brush was 95.6% and the specificity was 84.9% respectively (Table 2). This brush showed an overall diagnostic accuracy of 85.8%. Among the 1352 samples taken with the rigid brush, there were 89 cytological positive diagnoses. These included twelve false-positive diagnoses in the histological follow-up. 206 doubtful and suspicious findings included 31 malignant lesions in the comparison group. The cytological negative diagnoses resulted in a total value of 1057. Five false- negative diagnoses were revealed (Table 4).

**Table 4:** Modified 2 × 2 contingency table of brush biopsies using rigid brush compared to final histologically proven diagnoses

Cytology Test: rigid brush	Histology					
	Positive for malignancy		Negative for malignancy		Total	
Positive	77		12		89	
Suspicious	15	108	41	187	56	295
Doubtful	16		134		150	
Negative	5		1052		1057	
Total	113		1239		1352	
Overall accuracy	85.80%					

Since it is much easier to detect a large T3/ T4 lesion compared to a CIS or T1 lesion, we compared these lesions statistically. It can be seen that both brushes were also able to detect a relatively large number of SIN III/ CIS- lesions and T1- lesions or at least classified them as doubtful or suspicious (Table 5).

**Table 5:** Correlation matrix of cytological findings of both brushes compared to histology in 2018 cases of oral lesions

Cytology Test: nylon and rigid brush	Histology						
	Benign lesions	Mild dysplasia/ SIN I	Moderate dysplasia/ SIN II	Severe dysplasia/ SIN III/ CIS	Invasive SCC-T1	Invasive SCC-T > 1	Invasive SCC-Tx
Positive	13	0	1	7	29	79	14
Suspicious	44	1	1	0	8	13	9
Doubtful	164	0	0	1	8	8	5
Negative	1603	0	0	2	4	4	0
Sum	1824	1	2	10	49	104	28
Total	2018						

SIN- squamous intraepithelial neoplasia, SCC- squamous cell carcinoma, CIS- Carcinoma in situ

The risk suffering from a malignant oral lesion when the result of the brushes was positive, suspicious or doubtful was significantly high for both tests as shown by the odds ratio (nylon brush OR: 246.3; rigid brush OR: 121.5). To determine the statistical significance of the odds ratio, we chose the McNemar test and calculated *χ*^2^ (Table 2).

The average time for proper cell collection was measured in one hundred cases each test group. The mean time per case was 21 sec. (SD ± 0,3) for the rigid brush and 91 sec. for the nylon brush (SD ± 0,5). As a result, testing with the rigid brush was on average 4 times faster than with the nylon brush.

## Discussion

Early detection of oral squamous cell carcinomas is key to reducing mortality and morbidity rates [17]. Tumour size not only determines the type of curative therapy, but also influences its prognosis [8, 18]. With early tumour detection, the requisite therapy time can be reduced, offering the patient a considerable improvement in their quality of life. A significant loss of organ function as well as (visible) deformations can thus be avoided [19, 20]. There are two methods to increase early detection: First, visual and tactile screening can be used to diagnose patients, despite minor symptoms. Second, in addition to the previous method, specific oral diagnostic tools require further development. With early detection of oral SCCs, patients achieve an approximate five-year survival rate of 80% [19, 21, 22].

In this study, the brush biopsy technique was used for diagnostic sampling of conspicuous oral lesions. One major advantage of this procedure is that it does not require local anaesthesia and thus it is well accepted by most patients [19, 23, 24]. The brush has to be rotated ten times around its longitudinal axis with sufficient pressure against the lesion and adequate cell material must be removed in order to take several samples [25, 26]. All brush biopsies in this study were further processed liquid-based; no conventional smears, meaning it was directly transferred to glass slides were taken. The liquid-based brush biopsy technique has some advantages over the conventional method: current literature shows an improvement of 41% in cell thickness, while cell distribution is optimised by 66% [27]. The cell overlaps are highly reduced, and blood contamination is less frequent. Therefore, the cell morphology is more easily viewed and the cytopathological evaluation process streamlined. An easier chairside sample obtainment, storage and sample reproducibility are noticeable. Disadvantages of the liquid-based brush biopsy are higher costs for pathologists and a retention rate of 50% of the removed cell material [27].

Furthermore, Hayama et al. [27] showed that three of 44 (6.8%) conventional smears were hypocellular and therefore not interpretable. However, this did not occur with the liquid-based samples. In the past our research group published the results of 5328 conventional brush biopsies by direct transfer of the smear onto glass slides followed by an immediate alcohol fixation of the samples. The diagnostic accuracy achieved was a sensitivity of 97.17% and a specificity of 90.84%. In Remmerbach [9] at least five conventional smears were taken from each individual lesion and transferred to conventional glass slides chairside [28]. Navone et al. [29] compared both methods with regard to their sensitivity and specificity. The conventional method achieved a sensitivity of 85.7% and a specificity of 90.6%, while the liquid-based method was 95.1% and 99.0%.

In the present study, 2018 patient cases were evaluated. Sampling of conspicuous oral lesions was performed using liquid-based brush biopsies. After cytological evaluation, the diagnoses 'doubtful', 'suspicious' and 'positive' were summarized as 'tumour cell positive' and only the samples evaluated as 'negative' were recognized as 'tumour cell negative' for the statistical evaluation in comparison to histology [30].

A sensitivity of 95.6% and a specificity of 84.9% were achieved for the rigid brush. In comparison the nylon brush showed a sensitivity of 93.8% and a specificity of 94.2%. Using the rigid brush there were 187 patients diagnosed as false positive of altogether 1352 (13.83%). With the nylon brush, 34 patients were classified as false positive of altogether 666 (5.11%).

False positive diagnoses are a psychological burden for the patient and can mean unnecessary overtreatment. However, in the field of cancer diagnostics, false-negative diagnoses are of greater concern because it entails that a malignant transformation is overseen and was not treated early. This occurred in five out of 1352 cases (0.37%) with the rigid brush, compared to 5 out of 666 cases (0.75%) with the nylon brush.

Despite the decrease in specificity, we have chosen to prioritise the reduction of false negative diagnoses and maximise sensitivity by considering all non-negative results as ‘positive’; this includes both ‘doubtful’ and ‘suspicious’ results. This is in accordance with the binary biological behaviour of tissue cells: malignant or non-malignant. It was shown that in cases of regenerative epithelium, the cytopathologist tends to inflate the probability of malignant changes when the rigid brush is used, compared to the nylon brush. This necessarily leads to more ‘doubtful’ results being classified as false positives and thus a reduced specificity in this group.

In order to reduce the likelihood of false positive diagnoses, it is strongly recommended to use further adjuvant methods like DNA-image cytometry or Raman microspectroscopy [31–33]. The cytometric evaluation to determine the presence of DNA aneuploidy has a great influence on the early detection and therapy assessment of oral lesions and it is highly recommended [34].

Another promising adjuvant approach with a high diagnostic accuracy seems to be the cytology cell block technique including immunocytochemistry in the diagnosis of oral leukoplakia and oral squamous cell carcinomas [35, 36].

Certainly, the number of taken brushes per lesion influences the diagnostic accuracy: From 2009 on, five samples were taken and pooled in one SurePath™ vial with the use of the nylon brush, whereas the rigid brush required only one obtainment. These differing sampling methods influenced the sensitivity, as well as the specificity. The application of five nylon brushes takes more cells for the pathologist to examine than the single rigid brush, however, the repeated use of five brushes in daily routine is an unreasonable imposition on the patient. Overall, a single obtainment with the rigid brush provides sufficient diagnostic results, although the weakness in specificity may lead to more surgical biopsies.

A desirable future objective is the implementation of an internationally harmonised classification for oral cytology. This study shows the weakness of nationally implemented guidelines for extragenital cytology, which are incomparable to one another. Even the application of cervical classification models is, from our perspective, an auxiliary resp. stop-gap solution. The requested comparison of the cytological categories to the different grades of dysplasia remains a challenge. The restriction to two categories of oral dysplasia in the current WHO classification appears to be realistic [37–39].

This is, to the best of our knowledge the first study comparing different cell collectors and procedures in a clinical setting with a representative number of patients.

## Conclusion

The liquid-based brush biopsy is easy to use and takes very less time. It brings a wide diagnostic accuracy [19, 24, 40]. However, the optimal brush for oral liquid-based sampling should be used. The rigid brush is more effective and user-friendly in comparison to the nylon brush. It is also much easier to handle due to its geometric adaptation to oral anatomical conditions.

With 95.6% the rigid brush has a higher sensitivity than the nylon brush. Sensitivity is one of the most important parameters for early detection of oral cancer to generate medical safety. When looking at the statistical values, it is noticeable though that 11.1% doubtful diagnoses were made with the use of the rigid brush. In comparison, the nylon brush’s proportion of doubtful diagnoses is 5.4% which is only half of it. This different evaluation can influence the specificity and may be related to the fact that five samples were taken with the nylon brush.

Nevertheless, the specificity of the rigid brush still needs to be improved. For this purpose, the combined use of brush biopsy with additional adjuvant methods such as DNA-Image cytometry is strongly recommended [17].

## Data Availability

The datasets used and/or analysed during the current study are available from the corresponding author on reasonable request.

## Abbreviations

SCC: Squamous cell carcinoma
PMD: Potentially malignant disorder
WHO: World Health Organization
CVTE: Conventional visual and tactile examination
PAP: Papanicolaou
HPV: Human papillomavirus
GT: Gentle touch
CI: Confidence interval
OR: Odds ratio
